# Expression and prognostic significance of the polymeric immunoglobulin receptor in epithelial ovarian cancer

**DOI:** 10.1186/1757-2215-7-26

**Published:** 2014-02-26

**Authors:** Jonna Berntsson, Sebastian Lundgren, Björn Nodin, Mathias Uhlén, Alexander Gaber, Karin Jirström

**Affiliations:** 1 Department of Clinical Sciences, Division of Pathology, Lund University, 221 85 Lund, Sweden; 2 Science for Life Laboratory, Royal Institute of Technology, 171 21 Stockholm, Sweden; 3 School of Biotechnology, AlbaNova University Center, Royal Institute of Technology, 106 91 Stockholm, Sweden

**Keywords:** Polymeric immunoglobulin receptor, Fallopian tubes, Ovarian cancer, Prognosis

## Abstract

**Background:**

High expression of the polymeric immunoglobulin receptor (PIGR) has previously been associated with a favourable prognosis in a few cancer forms, but its expression and relationship with clinical outcome in epithelial ovarian cancer (EOC) has not yet been reported. The aim of this study was therefore to examine the clinicopathological correlates and prognostic significance of PIGR expression in EOC.

**Methods:**

After an initial screening in the Human Protein Atlas portal, a validated antibody was selected for extended analysis of immunohistochemical PIGR expression in tissue microarrays with tumours from 154 incident cases of EOC from two pooled prospective population-based cohorts. Subsets of corresponding benign-appearing fallopian tubes (n = 38) and omental metastases (n = 33) were also analysed. Kaplan-Meier analysis and Cox regression analysis were applied to examine the impact of PIGR expression on overall survival (OS) and ovarian cancer-specific survival (OCSS).

**Results:**

PIGR expression was significantly higher in fallopian tubes compared to primary tumours and metastases (p < 0.001) and lower in carcinoma of the serous subtype compared to other carcinomas (p < 0.001). PIGR expression was significantly associated with lower grade (p = 0.001), mucinous histological subtype (p = 0.002), positive progesterone receptor expression (p = 0.009) and negative or low Ki-67 expression (p = 0.003). Kaplan-Meier analysis revealed a significantly improved OS (p = 0.013) and OCSS (p = 0.009) for patients with tumours displaying high expression of PIGR. These associations were confirmed in unadjusted Cox regression analysis (HR = 0.48; 95% CI 0.26-0.87; p = 0.015 for OS and HR = 0.43, 95% CI 0.22-0.82; p = 0.011 for OCSS) but did not remain significant after adjustment for age, grade and clinical stage.

**Conclusions:**

This study provides a first demonstration of PIGR expression in human fallopian tubes, primary EOC tumours and metastases. High tumour-specific expression of PIGR was found to be associated with a favourable prognosis in unadjusted, but not in adjusted, analysis. These findings are novel and merit further investigation.

## Introduction

Epithelial ovarian cancer (EOC) is the fifth most common cancer type in women in more developed areas and the most lethal malignancy of the female reproductive tract [1]. In Sweden, EOC accounts for 3.1% of all cancers and 5.9% of all cancer deaths in women [2]. Due to vague symptomatology and the absence of reliable screening tests [3], the majority of EOC patients are diagnosed in advanced clinical stages, having stage III and IV tumours, with poor 5-year survival rates [4]. Hence, there is a need to identify novel diagnostic, prognostic and treatment predictive biomarkers.

Using the Human Protein Atlas (http://www.proteinatlas.org) as a tool for antibody based biomarker discovery [5], the polymeric immunoglobulin receptor (PIGR) was recently identified as being differentially expressed among EOC samples, with either negative or strong cytoplasmic and membranous staining. Thus, we hypothesized that PIGR might be a putative prognostic biomarker in EOC.

PIGR is a member of the immunoglobulin superfamily that binds polymeric immunoglobulin molecules at the basolateral surface of epithelial cells. The complex is then transcytosed across the cell to be modified and secreted at the apical surface as secretory component (SC) [6]. SC ensures effective mucosal secretion of polymeric immunoglobulins [6].

The clinicopathological significance of PIGR has hitherto only been investigated in a few studies. PIGR-negative adenocarcinomas in the distal oesophagus and gastroesophageal junction have been found to be more aggressive and to possess higher metastatic potential compared to adenocarcinomas with high expression of PIGR [7]. Low expression of PIGR in colorectal cancer was found to be associated with tumourigenicity [8] and with poor prognosis [9]. Furthermore, tumour progression in non-small cell lung cancer is reportedly associated with loss of PIGR expression [10]. One study reported associations between high expression of PIGR and type 1 endometrial cancer, suggesting a possible explanation for this less aggressive type [11]. On the contrary, overexpression of PIGR in hepatitis B-derived hepatocellular carcinoma has been described to correlate with higher metastatic potential and poor prognosis [12]. A study concerning bladder cancer lays forward a hypothesis that PIGR expression is associated with good prognosis, however, the study also points out the need for further research [13]. PIGR expression has not yet been described in EOC and consequently, this study will be novel.

The aim of this study was to evaluate the clinicopathological correlates and prognostic value, of PIGR expression in EOC, by immunohistochemical (IHC) analysis of 154 EOC samples from two pooled, prospective, population-based cohorts. The hypotheses of the study were that PIGR expression would differ in relation to histological subtype, and that a low expression of PIGR would be associated with poor prognosis.

## Material and methods

### Patients

The study cohort was a merge of all invasive EOC that had occurred in the two prospective population-based cohorts Malmö Diet and Cancer Study (MDCS, n = 101) [14] and Malmö Preventive Project (MPP, n = 108) [15] until December 31st 2007. The MDCS was initiated in 1991 and enrolled 17 035 healthy women [14,16], with main objective to obtain information about association between various dietary factors and cancer incidence [16]. The MPP was established in 1974 as a preventive case-finding programme for cardiovascular risk factors and enrolled 10 902 women [15].

Information on EOC incidence was obtained through the Swedish Cancer Registry up until December 31 2006, and from The Southern Swedish Regional Tumour Registry for the period of January 1 – December 31 2007. Thirty-five of the EOC patients participated in both studies, and archival tumour tissue could be retrieved from 154 (88.5%) of the total number of 174 cases. Information on clinical stage, following the standardised International Federation of Gynaecology and Obstetrics (FIGO) classification of tumour staging [17], and on treatment data was retrieved from medical charts. Histopathological data were obtained from pathology records. Tumours were divided into four groups according to histological subtype: serous (n = 90), endometrioid (n = 35), mucinous (n = 12) and others (n = 17). The latter group included clear cell (n = 9), Brenner (n = 1) and unknown (n = 7) tumours.

Standard surgical management of EOC patients consisted of total abdominal hysterectomy, bilateral salpingo-oophorectomy and omentectomy with cytological evaluation of peritoneal fluid or washings. Routine pelvic lymphadenectomy was not performed. Standard adjuvant therapy was platinum-based chemotherapy, from the mid 1990’s in combination with paclitaxel; however, treatment data was only available for 73 (47.4%) out of the total 154 cases and therefore not considered. Information regarding residual tumour after surgery was not available.

Median age at diagnosis was 62 (range 47-83). Information on cause of death in EOC cases was retrieved from medical charts and the Swedish Cause-of-Death Registry up until June 30 2012. Follow-up began at EOC diagnosis and ended at death, emigration or June 30 2012, whichever came first. After a median follow-up of 3.00 years (range 0–24.63), 122 patients (79.2%) were dead, 112 (72.3%) from ovarian cancer, and 32 (20.8%) were alive. The study cohort has also been described previously [18-22]. Ethical permission for the present study (Ref. 445/2007) was obtained from the Ethical Committee at Lund University. All patients gave written consent. Study design, methodological and technical considerations, as well as data presentation were based on the Reporting Recommendations for Tumor Marker Prognostic Studies (REMARK) criteria [23] (Additional file 1).

### Tissue microarray construction

All tumours were histopathologically re-evaluated and classified according to the WHO grading system of 2004 by a board certified pathologist (KJ). Tissue microarrays (TMAs) were constructed as previously described [21] using a semi-automated arraying device (TMArrayer, Pathology Devices, Westminister, MD, USA). In brief, two 1.0 mm cores were taken from viable, non-necrotic areas from all primary tumours (n = 154), when possible from different donor blocks, from matched fallopian tubes with no evidence of histological disease (n = 38) and peritoneal metastases (n = 33). All tumour samples were represented in duplicate tissue cores (1 mm).

### Immunohistochemistry

For immunohistochemical analysis, 4 μm TMA-sections were automatically pre-treated using the PT Link system and then stained in an Autostainer Plus (DAKO; Glostrup, Copenhagen, Denmark) with a polyclonal, monospecific antibody (HPA012012; Atlas Antibodies AB, Stockholm, Sweden) diluted 1:200. The specificity of the antibody has been confirmed by immunofluorescence, Western blotting and protein arrays (http://www.proteinatlas.org).

Analysis of immunohistochemical expression of androgen receptor (AR), estrogen receptor (ER), progesterone receptor (PR) and Ki67 was performed as previously described [20,21]. KRAS mutation status was analysed by pyrosequencing as previousy described [22].

### Evaluation of PIGR expression

IHC staining was annotated by two observers (JB, SL), blinded to clinical outcome, whereby consensus for each core was reached in estimated percentage groups as follows: 0 (0%), 1 (1-25%), 2 (25-50%), 3 (50-75%) and 4 (75-100%) stained cells. Staining intensity was annotated in groups of 0-3, whereby 0 = negative, 1 = weak, 2 = moderate and 3 = strong intensity. A multiplier of intensity and fraction, cytoplasmic score (CS), was calculated for each core and a mean value of the two cores was used in the analyses.

### Statistics

Mann-Whitney U test was used to assess distribution differences in continuous PIGR expression described by means of its median and range values in relation to clinicopathological characteristics and investigative factors.

Classification and regression tree (CRT) analysis was used to find the optimal cutoff value and the prognostic impact was also validated using ROC curve analysis.

Kaplan-Meier analysis and log rank test were used to analyse the difference in overall survival (OS) and ovarian cancer specific survival (OCSS) in relation to high and low PIGR expression as determined by CRT analysis. Cox regression proportional hazard models were used to estimate hazard ratios (HRs) for death from ovarian cancer or overall causes according to high and low PIGR expression in both uni- and multivariable analysis, adjusted for age, stage and grade.

All calculations were performed using SPSS version 21.0 (SPSS Inc, Chicago, IL). All statistical tests were two-sided and p-values < 0.05 were considered statistically significant.

## Results

### Distribution of PIGR expression in fallopian tubes, EOC and omental metastases

**Figure 1:**
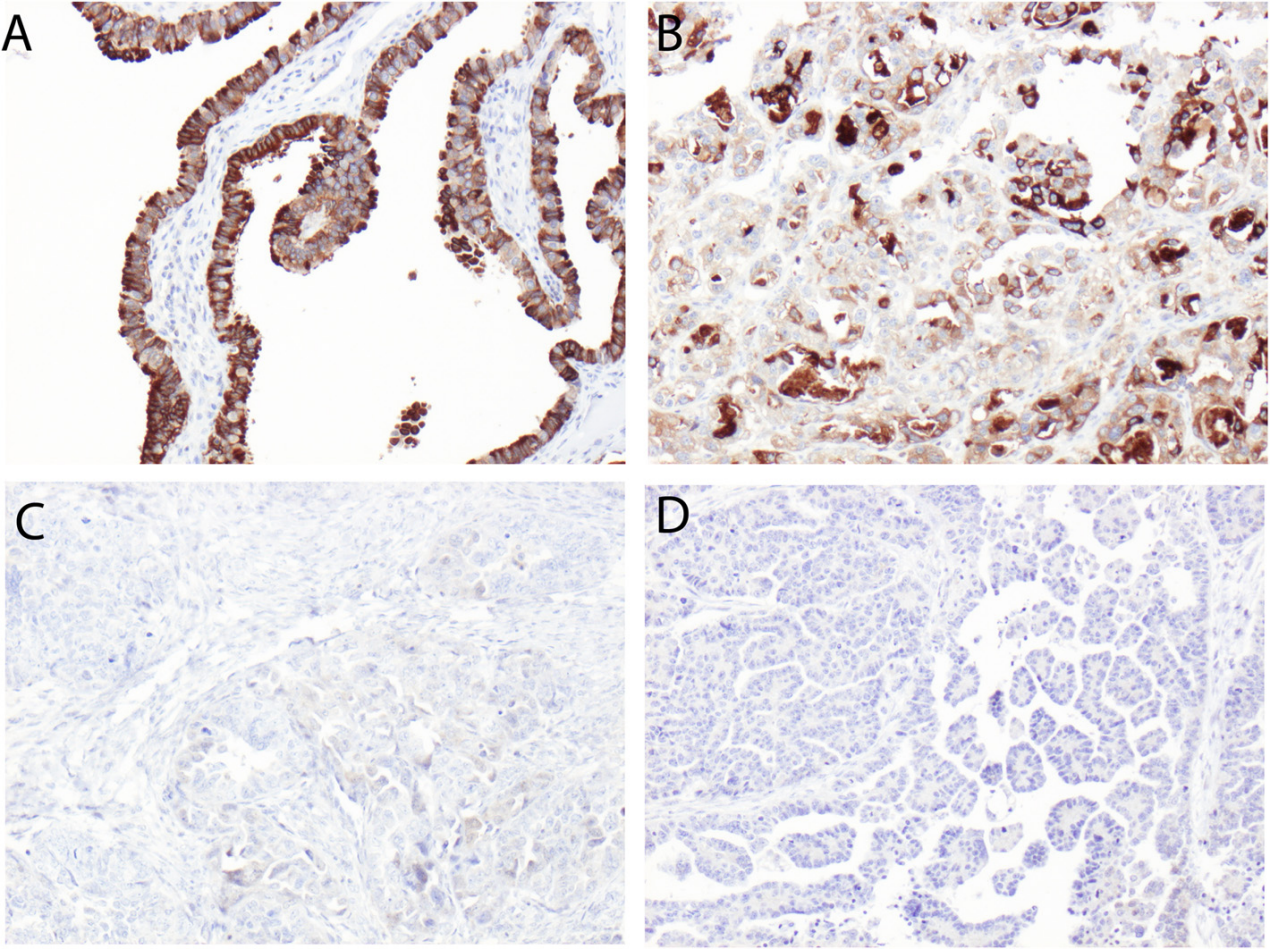
**Immunohistochemical images of PIGR staining in fallopian tubes, primary and metastatic epithelial ovarian cancer.** Sample images (10× magnification) representing immunohistochemical expression of PIGR, described as cytoplasmic score, i.e. a multiplier of fraction (0-4) and intensity (0-3) of staining: **(A)** Fallopian tube and **(B)** primary tumour with strong expression (score 12 and 12, respectively), **(C)** primary tumour with weak (score 3) and **(D)** matched metastasis with negative expression (score 0).

**Figure 2:**
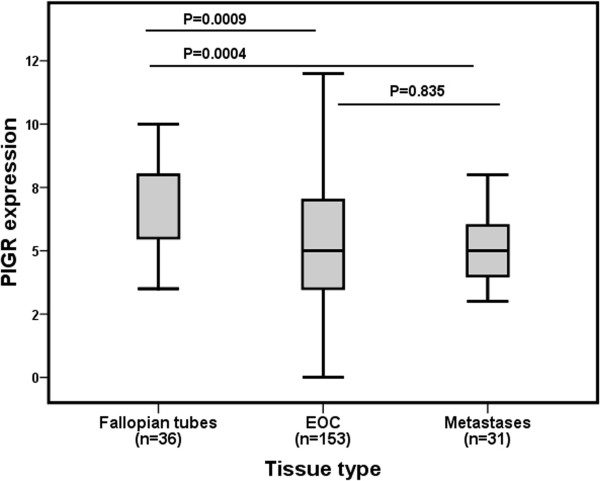
**Distribution of PIGR expressions in fallopian tubes, primary tumours and metastases.** Box plot visualising the staining distribution of PIGR in fallopian tubes, primary tumours and metastases. PIGR expression is described as cytoplasmic score, i.e. a multiplier of fraction (0-4) and intensity (0-3) of staining.

Thirty-six (94.7%) of the 38 sampled fallopian tubes were suitable for analysis and PIGR expression was evident in all cases, with a median CS of 8 (range 3.5 - 12). A total number of 153 (99.4%) primary tumours and 31/33 (93.9%) metastases were suitable for analysis, with a median CS of 5 (range 0 - 12) in the former, and 5 (range 0 - 8) in the latter. Sample images are shown in Figure 1. PIGR expression was significantly higher in fallopian tubes compared to primary tumours (p = 0.0009) and to metastases (p = 0.0004, Figure 2). There was no significant difference in PIGR expression between primary tumours and metastases (p = 0.835, Figure 2). As samples from all three locations were only available for six patients, this study did not allow for a meaningful analysis of PIGR expression related to individual tumour progression.

**Figure 3:**
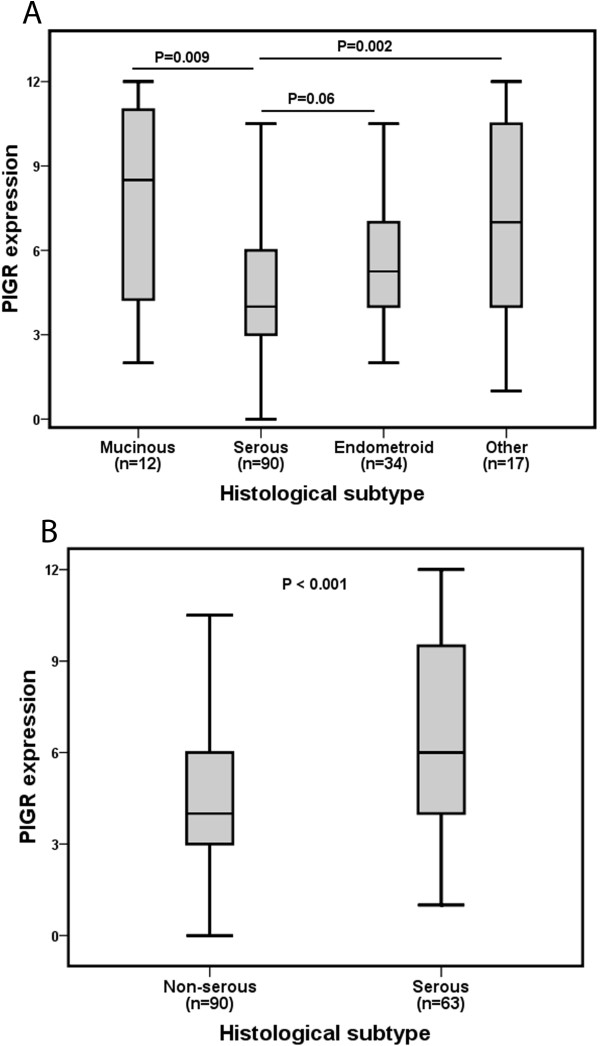
**Distribution of PIGR expression in histological subtypes.** Box plots visualising **(A)** the staining distribution of PIGR in different histological subtypes and **(B)** in serous vs non-serous carcinomas. PIGR expression is described as cytoplasmic score, i.e. a multiplier of fraction (0-4) and intensity (0-3) of staining.

PIGR expression was significantly lower in carcinomas of the serous subtype compared to the mucinous subtype (p = 0.009), and to other subtypes (p = 0.002, Figure 3A). A borderline significant difference in distribution was observed between serous and endometrioid tumours (p = 0.060, Figure 3A). PIGR expression was significantly lower in serous compared to non-serous carcinomas (p <0.001, Figure 3B).

The distribution of the total PIGR score in primary tumours is shown in Additional file 2.

### Associations of PIGR expression with clinicopathological characteristics

Associations between PIGR expression in primary tumours and established clinicopathological and investigative parameters are shown in Table 1. High PIGR expression was significantly associated with low grade (p = 0.001), mucinous histological subtype (p = 0.002), positive PR expression (p = 0.009) and negative or low Ki-67 expression (p = 0.003). Moreover, PIGR expression was borderline significantly associated with KRAS mutation (p = 0.061). No associations were found between PIGR expression and age, clinical stage or expression of AR or ER.

**Table 1:** Associations between PIGR expression and clinicopathological and investigative parameters

**Factor**	**PIGR expression**	**p-value**
	**median (range)**	
**Age**		
≤Median	4.00 (0.00–12.00)	0.258
>Median	5.00 (0.00–12.00)	
**Histological subtype**		
Serous	4.00 (0.00–10.50)	0.002
Endometroid	5.25 (2.00–12.00)	
Mucinous	8.50 (2.00–12.00)	
Other	7.00 (1.00–12.00)	
**Differentiation grade**		
Low	6.00 (0.00–12.00)	0.001
High	4.00 (0.00–12.00)	
**Clinical stage**		
I	7.00 (2.00–12.00)	0.084
II	5.00 (1.00–12.00)	
III	4.00 (0.00–12.00)	
IV	4.00 (0.00–12.00)	
**KRAS status**		
Wild–type	4.25 (0.00–12.00)	0.061
Mutated	6.75 (2.00–12.00)	
**Ki–67**		
0–10%	6.00 (1.00–12.00)	0.003
11–25%	4.00 (0.00–12.00)	
>25%	5.00 (0.00–12.00)	
**AR**		
Negative	5.00 (0.00–12.00)	0.674
Positive	4.00 (0.00–12.00)	
**ER**		
Negative	6.00 (0.00–12.00)	0.089
Positive	4.00 (0.00–12.00)	
**PR**		
Negative	4.00 (0.00–12.00)	0.009
Positive	6.50 (0.00–12.00)	

AR = androgen receptor, ER = oestrogen receptor, PR = progesterone receptor. The analysis of PIGR expression is based on multipliers of staining intensity and fraction of tissue stained (cytoplasmic score).

### Prognostic significance of PIGR expression

**Figure 4:**
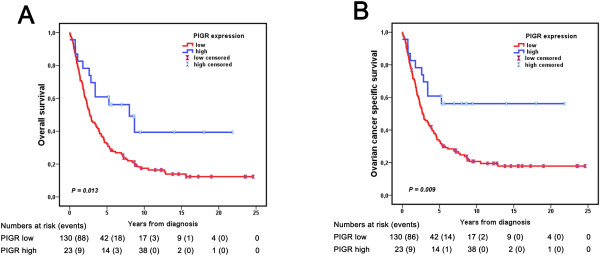
**Kaplan-Meier estimates of ovarian cancer specific and overall survival in all patients according to PIGR expression.** Kaplan Meier analysis of **(A)** overall survival and **(B)** ovarian cancer specific survival in strata of low and high PIGR expression. The categories of staining were determined by classification and regression tree analysis based on the cytoplasmic score (CS), whereby low expression = CS ≤ 8.5 and high expression = CS > 8.5.

CRT analysis established an optimal cutoff point at CS ≤ 8.5, which was used to stratify patients into groups of low (CS ≤ 8.5, n = 130) and high PIGR expression (CS > 8.5, n = 23), and the same prognostic cutoff was derived from ROC curve analysis (Additional file 3). Kaplan-Meier analysis of the entire cohort (n = 153) demonstrated a significantly prolonged OS (p = 0.013) and OCSS (p = 0.009) for patients with tumours displaying high PIGR expression (Figure 4). Univariate Cox regression analysis confirmed the relationship between high PIGR expression and a prolonged OS (HR = 0.478; 95% CI 0.263-0.868; p = 0.015) and OCSS (HR = 0.431; 95% CI 0.225-0.825; p = 0.011). However, these associations did not remain significant in multivariable analysis, adjusted for age, grade and clinical stage (data not shown). Analysis in strata according to histological subtype revealed that the prognostic impact of PIGR could not be attributed to a particular histological subtype (data not shown). Continuous PIGR expression was not significantly associated with clinical outcome (data not shown). Associations of high and low PIGR expression, defined by the CRT-derived cutoff , with clinicopathological factors were similar to comparisons of the distribution of the continuous PIGR score across categories (data not shown).

## Discussion

This study is, to the best of our knowledge, the first to investigate the expression, clinicopathological correlates and prognostic significance of PIGR in EOC. In addition, PIGR expression was evaluated in a subset of matched benign-appearing fallopian tubes and omental metastases.

The results demonstrate a significantly higher PIGR expression in fallopian tubes compared to primary and secondary tumour sites. Recent studies have suggested that a significant proportion of serous carcinomas arise within the fimbrial tubal epithelium [24,25]. Our findings indicate that malignant transformation could involve a downregulation of PIGR in EOC cases. PIGR expression in primary and metastatic tumours was rather similar, suggesting that downregulation of PIGR occurs early in ovarian carcinogenesis. Previous studies have suggested that omental spreading of EOC is due to wall shear stress in tumours as a result of fluid flow in the peritoneum or peristaltic movements [26,27], rather than from traditional metastasis-models such as haematogenous or lymphatic seeding, and thus, metastasising might not only occur due to tumour progression. This might explain why PIGR expression did not differ between primary and metastatic tumours. However, as this study only included six patients with samples from all three locations, further studies are needed to analyse PIGR expression related to individual tumour progression.

There were no significant associations between PIGR expression and clinical stage or age, which is in line with previous studies in oesophageal [7], bladder [13] and non-small cell lung cancer [10]. PIGR expression was found to be significantly associated with positive PR status, negative or low Ki-67 expression and borderline significantly associated with KRAS-mutation. High expression of Ki-67 has previously been described to correlate with more advanced tumour stage and peritoneal spread [28], whereas KRAS mutation reportedly is associated with well-differentiated EOC tumours [22]. Lee *et al.* reported PR to be an independent predictor of good prognosis [29]. However, another study analysed PR status in strata according to different histological subtypes, whereby no correlation with prognosis was found [21]. Nevertheless, the findings of the present study suggest an association between high PIGR expression and a less malignant phenotype, reflected in the associations with the described clinicopathological parameters as well as a more favourable clinical outcome

Similar associations have previously been described in studies on PIGR expression in oesophageal [7], colorectal [9], non-small cell lung [10], endometrial [11] and bladder cancer [13].

Another plausible explanation for the favourable prognosis in patients with tumours displaying high expression of PIGR has been suggested. One study speculated that overexpression of PIGR may be part of the host’s response to the presence of cancer cells or to carcinogenic stimulus [11]. This explanation originates from the fact that SC, a cleaved form of PIGR, is a known inhibitor of proinflammatory cytokine IL-8 and, as a consequence, of polymorphonuclear neutrophils (PMNs) [11]. Although PMNs are generally accepted as being antitumorigenic [11], Dong *et al.* reported PMNs facilitating extravasation of melanoma cells [30]. The study also described a reduced tumour extravasation by IL-8 receptor-blocking or neutralisation of soluble IL-8 [30]. Additionally, PMNs have been described to promote tumour progression by activating matrix metalloproteinase-2 (MMP-2), a proteinase involved in angiogenesis, tumour invasion and metastasis [11,31]. Thus, high levels of SC may reduce the occurrence of metastases and prevent tumour-induced angiogenesis and tumour invasiveness. However, studies on colorectal [32] and hepatocellular [33] cancer suggest that SC serum levels are not necessarily associated with tumour-specific PIGR expression and the described association might therefore not be applicable for the present study.

A limitation to this study is the lack of information on residual tumour after surgery, however, as PIGR expression did not provide any independent prognostic value, inclusion of this information in the multivariable model is not likely to have altered our findings. Future studies of PIGR expression in EOC should nonetheless, when possible, incorporate this factor in multivariable models.

Another limitation was the subjective nature of IHC staining assessment. In order to avoid bias, the TMAs were not sorted by differentiation or histological subtype. The observers were blinded to clinical outcome, and any scoring differences were discussed in order to reach consensus. To further decrease the impact of subjectivism, image analysis software could have been an option [34]. However, the specificity and sensitivity of automated software are still unclear [35] and therefore, a semiquantitative assessment strategy was deemed more appropriate. Tissue heterogeneity might also pose a difficulty [34,36], however, to increase representativeness and decrease influence of heterogeneity, one should include several samples from the same tissue [35,37], as was done in this study.

While PIGR expression was found to be significantly lower in tumours of the serous subtype compared to non-serous tumours, its prognostic value did not differ across subtypes. Of note, the number of some histological subtypes was rather limited in the here studied cohort and, therefore, future studies on the prognostic value of PIGR expression in EOC should ideally include a larger number of samples from different subtypes. It will also be of interest to examine PIGR expression in relation to the recently introduced complementary classification system, wherein EOC is divided into the less aggressive type I and more malignant type II tumours, corresponding to two potential main pathways of tumourigenesis, that differ with respect to mutation pattern and prognosis [38].

## Conclusions

In this pooled, prospective population-based cohort of epithelial ovarian cancer, significant associations were found between PIGR expression and mucinous histology, low-grade tumours and Ki-67 expression, indicating a less aggressive phenotype for tumours displaying high PIGR expression. Moreover, patients with high tumour-specific expression of PIGR had a significantly prolonged survival in unadjusted analysis, but not when adjusted for age, grade and clinical stage. These results are novel, and merit further study in a functional context as well as in additional patient cohorts.

## Abbreviations

AR: Androgen receptor; CRT: Classification regression tree; CS: Cytoplasmic score; EOC: Epithelial ovarian cancer; ER: Oestrogen receptor; FIGO: International Federation of Gynaecology and Obstetrics criteria; HPA: The Human Protein Atlas; HR: Hazard ratio; IHC: Immunohistochemical; MDCS: Malmö Diet and Cancer Study; MMP-2: Matrix metalloproteinase-2; MPP: Malmö Preventive Project; OCSS: Ovarian cancer specific survival; OS: Overall survival; PIGR: Polymeric immunoglobulin receptor; PMN: Polymorphonuclear neutrophils; PR: Progesterone receptor; PrEST: Protein epitope signature tags; SC: Secretory component; TMA: Tissue microarray.

## Competing interests

The authors declare that they have no competing interests.

## Authors’ contributions

JB and SL evaluated the immunohistochemical stainings, performed the statistical analyses and drafted the manuscript. BN constructed the TMAs and performed the immunohistochemcal stainings. MU contributed with antibody validation. AG assisted with the statistical analysis and helped draft the manuscript. KJ conceived of the study, evaluated the immunohistochemistry, and helped draft the manuscript. All authors read and approved the final manuscript.

## Supplementary Material

Additional file 1 Reporting recommendations for REMARK and descriptions of how criteria are fulfilled. Click here for file

Additional file 2 **Distribution of PIGR staining in primary EOC.** Full range of the cytoplasmic score (intensity x fraction) of PIGR in primary tumours from 153 cases. Click here for file

Additional file 3 **Classification regression tree (CRT) and ROC curve analysis for selection of prognostic cutoffs according to PIGR expression.** CRT analysis of **(A)** overall survival and **(B)** ovarian cancer-specific survival, and **(C)** ROC curve analysis of overall survival based on the total score of PIGR expression in 153 primary tumours. Click here for file
