# Supplementary material for: Expression and prognostic significance of the polymeric immunoglobulin receptor in epithelial ovarian cancer
Source: J Ovarian Res. 2014 Feb 26;7:26. doi: 10.1186/1757-2215-7-26 (PMC3938822; doi:10.1186/1757-2215-7-26)
Supplement: Additional file 1 — Reporting recommendations for REMARK and descriptions of how criteria are fulfilled. [file 1757-2215-7-26-S1.doc]

# Additional File 1.

# Reporting recommendations for REMARK and descriptions of how criteria are fulfilled.

| **Reporting recommendations for REMARK** | **How criteria are fulfilled** |
| --- | --- |
| **Introduction** |  |
| 1. State the marker examined, the study objectives, and any pre-specified hypotheses | The hypothesis and study objectives are stated on p 4. |
| **Materials and Methods** |  |
| *Patients* |  |
| 1. Describe the characteristics of study patients, including their source and inclusion and exclusion criteria. | The characteristics of the study patients are given on p 5-6, with references. Inclusion and exclusion criterias are stated on p 5-6. This is a population based, prospective cohort study. Details of the study population are described on pp 5-6. |
| 2.Describe treatments received and how chosen | Treatments received are described on page 6. |
| *Specimen Characteristics* |  |
| 1. Describe type of biological material used and methods of preservation and storage | The study is performed on archival paraffin-embedded tumour material, from which suitable specimens were assembled in tissue microarrays, all stored and handled in room temperature. Described on p 6. |
| 2. Specify the assay used and provide (or reference) a detailed protocol, including specific reagents or kits used, quality control procedures, reproducibility assessments, quantitation methods, and scoring and reporting protocols. Specify whether and how assays were performed blinded to the study endpoint. | Details of assays and protocols used are given under Methods pp 6-7.  Assays were performed blinded to the study endpoint. |
| *Study Design* |  |
| 1. State the method of case, selection including whether prospective or retrospective and whether stratification or matching was used. Specify the time period from which cases were taken, the end of the follow-up period, and the median follow-up time. | This is a population based, prospective cohort study. Details of case selection and the time period are described on pp 5-6. |
| 2. Precisely define all clinical endpoints examined. | Details of endpoints are given on p 6 . |
| 3. List of all candidate variables initially examined for inclusion in models. | Described on p 7. |
| 4. Give rationale for sample size; if the study was designed to detect a specified effect size, give the target power and effect size. | Not applicable. |
| *Statistical analysis methods* |  |
| 5. Specify all statistical methods, including details of any variable selection procedures and other model-building issues, how model assumptions were verified, and how missing data were handled. | Specified on p 8. |
| 6. Clarify how marker values were handled in the analysis. | Clarified on pp 7-8. |
| **Results** |  |
| *Data* |  |
| 1. Describe the flow of patients through the study, including the number of patients included in each stage of the analysis and reasons for drop out. Specifically, report the number of patients and the number of events. | This was not a staged analysis. The evaluated cohort is described on pp 5-6. |
| 2. Report distributions of basic demographic characteristics, standard prognostics variables | These are described in the Material and Methods section pp 5-6 and also in several references to previous studies based on this cohort |
| *Analysis* |  |
| 1- Show the relation of the marker to standard prognostic variables | Table 1 and Figure 3 shows correlations between the investigated marker and standard prognostic variables. Standard prognostic indicators are also included in the multivariate analyses, as described on p 8. |
| 2. Present univariate analyses showing the relation between the marker and outcome, with the estimated effect (e.g., hazard ratio and survival | Presented in Figure 4 and in the Results section p 10 |
| 3. For key multivariable analyses, report estimated effects (e.g., hazard ratio) with confidence intervals for the marker and, at least for the final  model, all other variables in the model | The prognostic value of established clinicopathological factors has been demonstrated in previous papers, refered to here. |
| 4. Among reported results, provide estimated effects with confidence intervals from an analysis in which the marker and standard prognostic  variables are included, regardless of their statistical significance | The lack of prognostic significance for the investigative marker is described in the Results section p 10. |
| 5. If done, report results of further investigations, such as checking assumptions, sensitivity analyses, and internal validation | Not applicable |
| Discussion |  |
| 1. Interpret the results in the context of the pre-specified hypotheses and other relevant studies; include a discussion of limitations of the study | Discussed on pp 11-12 |
| 2. Discuss implications for future research and clinical value | Discussed on p 12 |
