# Supplementary figures and images for: Expression and prognostic significance of the polymeric immunoglobulin receptor in epithelial ovarian cancer
Source: J Ovarian Res. 2014 Feb 26;7:26. doi: 10.1186/1757-2215-7-26 (PMC3938822; doi:10.1186/1757-2215-7-26)

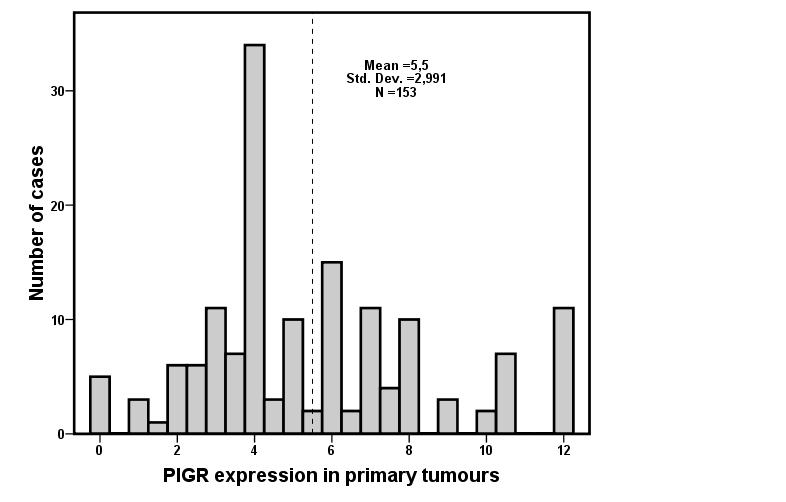

Supplement: Additional file 2 — Distribution of PIGR staining in primary EOC. Full range of the cytoplasmic score (intensity x fraction) of PIGR in primary tumours from 153 cases. [file 1757-2215-7-26-S2.jpeg]

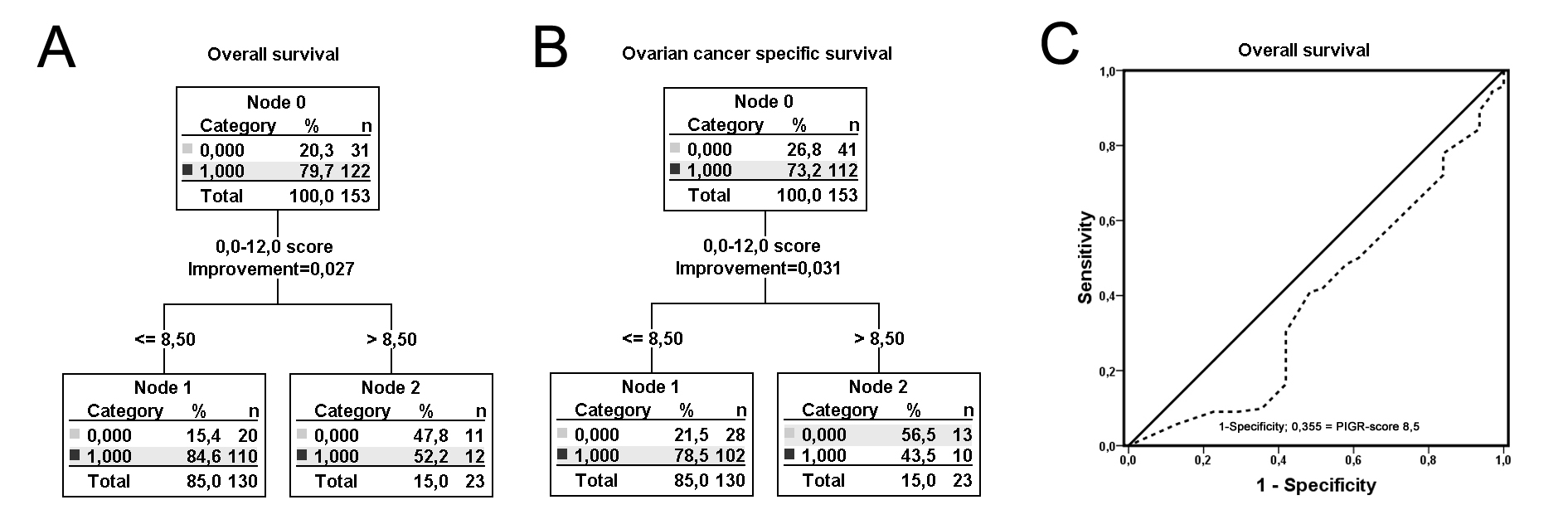

Supplement: Additional file 3 — Classification regression tree (CRT) and ROC curve analysis for selection of prognostic cutoffs according to PIGR expression. CRT analysis of (A) overall survival and (B) ovarian cancer-specific survival, and (C) ROC curve analysis of overall survival based on the total score of PIGR expression in 153 primary tumours. [file 1757-2215-7-26-S3.jpeg]
